# How Shall the Practitioner Continue His Education?1Read as part of Committee on Intelligence and Education’s Report at the Annual Convention of the United States Veterinary Medical Association, at Des Moines, Iowa, September, 1895.

**Published:** 1896-03

**Authors:** R. A. Archibald

**Affiliations:** Member of Committee


### HOW SHALL THE PRACTITIONER CONTINUE HIS EDUCATION?[1]

By Dr. R. A. ARCHIBALD,
MEMBER OF COMMITTEE.

For the benefit of the young veterinarian who is endeavoring to gain an enviable position as a successful practitioner I wish to make a few suggestions that, to a certain extent, may be foreign to the question under consideration. and at the same time may not directly pertain to veterinary science; still, in my estimation, a knowledge of these matters is very essential for the practitioner to acquire in connection or on top of his college education. For convenience I propose to deal with this matter under three heads: First, the practitioner should learn to do his duty to his clients; second, his duty to his colleagues; and, third, his duty to himself.

The veterinary practitioner is of necessity thrown in contact with all classes and varieties of people, and it is necessary for

[1] Read as part of Committee on Intelligence and Education's Report at the Annual Convention of the United States Veterinary Medical Association, at Des Moines, Iowa, September, 1895.

him to do his duty under many and varying circumstances. It will be seen, therefore, the necessity of studying human nature in its various forms, moods, and peculiarities, so he may be prepared to appreciate and encourage human virtues, and meet with good temper, tact, and success, human frailities in all their many degrees and phases. The treatment of the client is just as important, and in many instances more important, than the treatment of the patient. It is absolutely necessary for him to take a deep interest in his patients; he should avoid if possible considering his patients as mere cases of disease or injury, and by showing an interest and acuteness of feeling and sensibility for the patient he will naturally in the majority of instances retain the confidence of his client. In all connections with his client he should act upon general honest principles, and should exercise tact and discretion in recommending treatment which his knowledge teaches him to be right, more especially in cases where treatment is unnecessary; for if the practitioner does not exercise some considerable tact and patience in connection with the treatment of an imaginary disease, the result would be that his client would place the case in the hands of some unscrupulous parasite, who might, by encouraging the weakness and peculiarities of the owner, succeed in lowering the reputation of the practitioner in that community. It will be readily seen that the requirements of a knowledge of the proper method of dealing with different clients is by no means the least important matter which the practitioner must strive to attain.

We will now consider the practitioner's duty to his colleagues. There is generally a feeling among young practitioners against consultations, either private or public. It is necessary for them to learn the error of such a view. There is no greater mistake a practitioner can make than to decline consulting with a fellow-practitioner when a difficulty occurs in a case or when a client becomes impatient. Young practitioners are generally apt to think that when a consultation is requested that it denotes a lack of confidence in either the client or the attending practitioner himself, when very probably such is not the case; but whether it be or not they will best forward their own interests by cheerfully agreeing to a consultation. It is possible that a consultation may involve a difficult and delicate question sometimes, as to the honest expression of opinion in reference to the real condition of a patient, the exact cause of disease, etc.; but these matters can be properly and safely adjusted by the exercising of good, thoughtful judgment, and as a result confidence is restored to the mind of the client. Of course, where a consultation is requested it will be policy for the attending practitioner to endeavor to have his client procure the services of a professional man who is versed upon professional ethics.

A practitioner's duty to himself is the most important matter of all upon which he should educate himself. It consists chiefly in retaining his self-respect. Every practitioner has knowledge pertaining to his patients which is of a strictly private character, and except in cases where dishonesty is intended on the part of the client, no practitioner is justified in disclosing them. He should beware of gossiping in connection with his practice. Gossip may be all very well in social matters, but it should be strictly excluded from all matters of a professional nature. Frequently inquisitive individuals will try to extract information regarding the condition of a patient, etc. The practitioner in such a case should positively refuse to discuss such questions.

Punctuality is a habit that can and should be acquired by the practitioner who hopes to make a success. We have known of several instances where the practitioner has lost that lucky chance which is not unfrequently the beginning of future success.

Be courteous. Few men are more tried than members of the veterinary profession. We realize that human nature under all circumstances in which the veterinarian is placed cannot always command a sweet and unruffled temper, but he should learn to do his best in order to retain his self-respect. There is a popular idea that veterinarians are continually quarrelling among themselves. This idea is usually an exaggerated one, but, unfortunately, there are some grounds for it. Two causes appear to act as exciting influences in producing professional disputes. One is that clients too frequently forget the services of their veterinarian and in their anxiety, or in search of novelty, rush from one practitioner to another without proper consideration for their veterinarian's feelings. The other is that in some localities professional competition is very active, and where many are struggling for work and existence friction becomes serious. The practitioner, in order to retain his self-respect, should conform strictly to the rules laid down under the heading of professional etiquette, which is nothing more or less than the Golden Rule: "Do unto others as he would that they should do unto him."

The veterinarian should be always ready to assist a brother practitioner, providing he is not outside the proper professional standard. Veterinarians, like other individuals, will receive insults, but in the majority of cases the practitioner and the individual have themselves to blame for it. The practitioner should learn to know when the limits of professional endurance are passed and when it is becoming for him to insist upon a proper recognition of himself and his profession.

Whatever difficulties and anxieties the practitioner may meet with in practising his profession, he should bear in mind that the highest and noblest aim of his profession is to assist and relieve the suffering animal. In carrying out this high principle he may be imposed upon, and he may meet with ingratitude and insult, but he can rest assured that if he practises his profession in a thoroughly honest and conscientious spirit he will receive in return demonstrations of the finest feeling of human nature and tokens of truly sincere gratitude which will more than counteract his unpleasant experiences.

All this and more will the young practitioner have to add to the store of knowledge gained at college before he can lay claim to the enviable position of a successful veterinarian.

